# Timing of post contrast T1 values affects the extracellular volume fraction in ST segment elevation myocardial infarction

**DOI:** 10.1186/1532-429X-17-S1-P126

**Published:** 2015-02-03

**Authors:** Elisa McAlindon, Chris B Lawton, Chiara Bucciarelli-Ducci

**Affiliations:** Bristol Heart Institute, Bristol, UK; Heart and Lung Centre, New Cross Hospital, Wolverhampton, UK

## Background

Native T1 is increased in acute myocardial infarction. Extracellular volume fraction (ECV) can be calculated using T1 mapping following contrast. The timing of imaging following contrast in acute myocardial infarction is not established. 15 minutes has been proposed for conditions with and ECV < 0.4. 15 minutes may not be applicable in acute myocardial infarction due to infarct characteristics (ie MVO) impacting on contrast pharmacokinetics and the relatively higher ECV. The aim of this study was to determine if ECV is affected by the timing of imaging for post contrast T1 maps in STEMI.

## Methods

30 consecutive patients presenting with ST segment elevation myocardial infarction to the Bristol Heart Institute Primary PCI service were approached for inclusion in the study. Patients had a CMR scan day 2 following STEMI. Native T1 was assessed on T1 mapping (MOLLI, WIP Siemens). Post contrast T1 mapping was performed at 15 and 25 minutes following contrast administration. ECV was calculated as: Myocardial ECV = (1−hematocrit) × (ΔR1myocardium/Δ R1blood), where R1 = 1/T1. The difference in ECV at both time points was assessed by paired t-test. All patients gave written informed consent. The study was approved by the local ethics committee.

## Results

**Figure 1:**
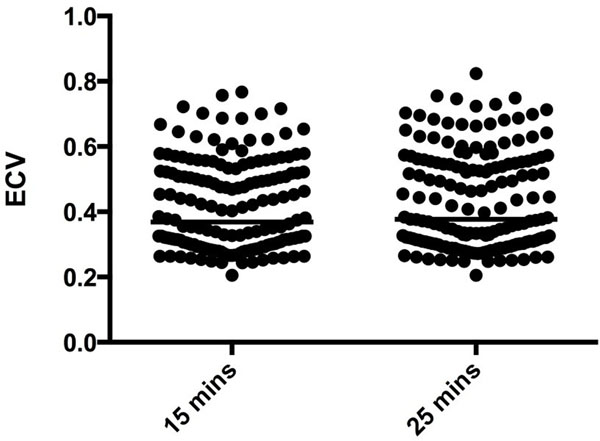
**The difference between T1 values 15 and 25 minutes following cintrast administration.** There is a significant difference between T1 values at 15 mins and 25 mins following contrast administration p<0.0001.

There was good correlation of ECV calculated from post contrast T1 mapping at 15 and 25 minutes p<0.0001 (r^2^=0.90, r=0.95). However, there is a significant difference between ECV calculated at 15 minutes (0.37, IQR 0.30-0.52) and 25 minutes (0.38, IQR 0.30-0.55), p<0.0001(Figure 1).

## Conclusions

In acute myocardial infarction, timing of post contrast T1 mapping affects ECV calculation.

## Funding

This study was funded by the National Institute for Health Research Biomedical Research Unit in Cardiovascular Disease at the University Hospitals Bristol NHS Foundation Trust and the University of Bristol.

